# The association between blood manganese and liver stiffness in participants with chronic obstructive pulmonary disease: a cross-sectional study from NHANES 2017–2018

**DOI:** 10.1186/s40001-022-00977-5

**Published:** 2023-01-07

**Authors:** Kexing Han, Jiapei Shen, Kexuan Tan, Jiaying Liu, Weijie Sun, Yufeng Gao

**Affiliations:** Department of Infectious Diseases, The First Affiliated Hospital of Anhui Medical University, No. 218 Jixi Road, Shushan District, Hefei, 230022 China

**Keywords:** Chronic obstructive pulmonary disease, Blood manganese, Liver stiffness, Cross-sectional study, Strengths and limitations of this study

## Abstract

**Background:**

To explore the relationship between blood manganese and liver stiffness in the United States among participants with chronic obstructive pulmonary disease (COPD).

**Methods:**

All data were obtained from the 2017–2018 National Health and Nutrition Examination Survey database (NHANES). A total of 4690 participants were included in the study. All participants included complete information on COPD, liver stiffness, and blood manganese. Liver stiffness (kPa) was measured from “Examination Date” and blood manganese (ug/L) was obtained from “Laboratory Data”. A multiple linear regression model was used to assess the correlation between blood manganese and liver stiffness.

**Results:**

Among the 4690 participants, blood manganese was lower in the COPD group but liver stiffness was higher (*p* < 0.05). There was a positive correlation between blood manganese and liver stiffness (*β* = 0.08, 95% CI 0.03, 0.12). This positive association was more pronounced in COPD participants (*β* = 0.25, 95% CI 0.08, 0.42) and there was a non-linear relationship, which was more significant when blood manganese exceeded 14.43 ug/L (*β* = 1.76, 95% CI 1.10, 2.41).

**Conclusions:**

The association between blood manganese and liver stiffness was positive, which was more apparent in COPD patients.

## Strengths and limitations of this study

1. Liver stiffness and blood manganese in patients with chronic obstructive pulmonary disease (COPD) are rarely concerned but important indicators in clinical work. This paper first explored the association between blood manganese and liver stiffness in patients with COPD.
2. The important cut-off points in the positive association between blood manganese and liver stiffness in COPD patients were found.
3. Discovered the sensitive crowds of association between blood manganese and liver stiffness for COPD and non-COPD participants
4. which could provide some reference for public health work.
5. There might be some deviation in the determination of COPD participants
6. self-assessment of dietary health status
7. and recalled smoking history in the study. At the same time
8. the sample size of COPD patients in subgroup analysis might be limited to a certain extent

## Introduction

As one of the essential trace elements [1], manganese is present in most tissues and plays an important role in maintaining intracellular homeostasis and normal growth and development of the human body, and insufficient or excessive manganese can lead to serious consequences [2]. For example, manganese ions are the active centre of manganese superoxide dismutase in the body, which has the ability to digest harmful free radicals, but can be counterproductive when overexposed [3]. Manganese exposure is a global environmental problem. Manganese is present in the natural environment, while anthropogenic sources such as mining and industrial production can increase the release of manganese, resulting in manganese contamination of the environment, including air and water. While there are identified high risk groups such as industrial workers, the general population is also being threatened by manganese exposure. In the human body, the main site of manganese metabolism is the liver [4]. Excessive intake of manganese can overload the liver [5], which can lead to liver diseases such as liver fibrosis and even damage to other organs. Previous study found that mean blood manganese levels were significantly higher in patients with cirrhosis than in people without cirrhosis [6]. Meanwhile, individuals with hepatic encephalopathy or liver failure are more likely to retain more manganese in their bodies [7]. There were also numerous previous studies on neurotoxicity due to manganese ion accumulation [8]. Recently, there has been an increasing interest in the effect of manganese ion levels in the blood on the respiratory system [9]. Therefore, it is particularly important to explore the relationship between blood manganese levels and the development and progression of related diseases.

Chronic obstructive pulmonary disease is a typical heterogeneous disease closely related to environmental factors, and its onset and acute exacerbations are associated with long-term chronic exposure to fine particulate matters (e.g., PM2.5) [10]. A recent study has shown that the inorganic component of PM2.5 in human blood is associated with the pathogenesis of COPD [11]. Oxidative stress is an important feature of the pathogenesis of COPD and has a significant impact on several factors of lung physiology and COPD pathogenesis [12]. Excess manganese induces oxidative stress, exacerbates mitochondrial dysfunction, dysregulates autophagy, promotes apoptosis, and leads to neurodegeneration [1]. Therefore, it is of great interest to measure serum manganese indicators in people with COPD. Meanwhile, COPD eventually develops into chronic pulmonary heart disease, which in turn causes pulmonary hypertension, right ventricular enlargement leading to liver congestion and even liver fibrosis. Therefore, liver stiffness is also of clinical value for monitoring COPD patients.

Few studies have examined the relationship between blood manganese and liver stiffness in specific populations. Liver transient elastography (LUTE) is a non-invasive method that is now widely used to detect liver fibrosis [13]. NHANES is a research program designed to assess the health and nutritional status of adults and children in the U.S. Therefore, we conducted a cross-sectional study to explore the association between blood manganese in the COPD population by analyzing data from NHANES 2017–2018 and correlation between liver stiffness.

## Methods

### Study design and setting

The NHANES database is widely used for cross-sectional studies, which includes participant demographic, nutritional, health status, physical examination, and laboratory data and is updated every 2 years. NHANES 2017–2018 was selected for this study because it included complete blood manganese, transient liver elastography, and COPD information for participants. In addition, the ethical review of this study was exempted because the database is accessible to the public.

### Participants

A total of 9254 participants were sampled for NHANES 2017–2018, of which those who participated in the question " Ever told you had COPD?" were limited to no less than 20 years of age. A total of 5569 participants took part in the questionnaire, of which 697 had no liver transient elastography data, 173 had no blood manganese data, 7 had no information on diet status and 2 had no information on smoking. The final 4690 participants were included in this study.

### Variables

Patients with COPD, congestive heart failure (CHF) and liver conditions were identified from the results of questionnaires on medical conditions, including 'Ever been told you had COPD?', 'Ever been told you had congestive heart failure?' and 'ever been told that you have liver condition?'. Information for participants with diabetes was obtained from the 'Diabetes' questionnaire with the question 'Doctor told you have diabetes?'. Median liver stiffness data for the primary outcome indicator were obtained from the examination date, which included complete liver transient elastography information. Serum manganese (ug/L) data were obtained from laboratory test.

Information on smoker (smoked at least 100 cigarettes in life), alcohol drinking (drinking alcohol more than 2 times per week in the past 12 months), diet status (answers to the question 'How healthy is the diet?'), prescription medications used (taken prescription medicine, past month) was collected from the questionnaire date. Age, gender, race, and PIR (income to poverty ratio) were obtained from demographic data.

### Statistical analysis

All data collection and analysis were performed through R (http://www.R-project.org) and EmpowerStats (http://www.enpowerstats.com), and *P* < 0.05 was considered statistically significant. To make the participants more representative, 2-year sampling weights were adopted in the analysis. Continuous variables were expressed as mean ± standard deviation, and when the missing sample size was less than 12%, the mean was used instead. Otherwise, continuous variables were set as "unclear group" after grouping. Categorical variables were represented as percentages, and missing data were defined as 'Unclear group'. Association between blood manganese and liver stiffness was analyzed by multiple linear regression model. Covariates included: age, sex, race, BMI, PIR, smoking, alcohol consumption, dietary status, prescription medication use, COPD, diabetes, congestive heart failure, and liver condition. According to different adjustment variables, Model 1: no adjustment model, Model 2: adjustment of age, gender and race, and Model 3: full adjustment model are generated. Subsequently, we also performed a subgroup analysis of the association between blood manganese and liver stiffness in both groups to find sensitive crowds. To explore the non-linear relationship between blood manganese and liver stiffness, we performed a smooth curve fitting model analysis and excluded significant blood manganese outliers. We also generated threshold effect models to find valid blood manganese cut-off points.

## Results

A total of 4690 participants were included in this study, and the analysis of general characteristics about the participants was shown in Table 1. The two groups differed in age, race, PIR, smoking history, prescription drug use, and dietary status (*P* < 0.05), and participants with COPD had a higher prevalence of comorbid diabetes, congestive heart failure, and liver disease (*P* < 0.05). However, there was no statistical difference between the two groups in terms of frequency of alcohol consumption (*P* = 0. 12).

**Table 1:** Characteristics of participants

Characteristics	COPD (*n* = 223)	Non-COPD (*n* = 4467)	*P* value
Age (years)	65.05 ± 11.54	50.50 ± 17.39	< 0.01
Striated by age (years) (%)			< 0.01
< 60	28.25	63.96	
≥ 60	71.75	36.04	
Gender (%)			0.44
Male	51.57	48.89	
Female	48.43	51.11	
BMI (kg/m^2^)	30.01 ± 8.72	29.74 ± 7.14	0.71
Striated by BMI (kg/m^2^) (%)			0.54
< 28	47.53	45.42	
≥ 28	52.47	54.58	
Race (%)			< 0.01
White	70.85	57.09	
Black	16.59	23.10	
Other race	12.56	19.81	
PIR (%)			< 0.01
< 1.39	45.74	25.45	
1.39–3.49	32.29	34.03	
> 3.49	10.76	27.83	
Unclear	11.21	12.69	
Alcohol drinking (%)			0.12
Yes	11.21	11.06	
No	78.48	73.63	
Unclear	10.31	15.31	
Smoker (%)			< 0.01
Yes	14.80	60.0	
No	85.20	40.0	
Diet status (%)			< 0.01
Excellent	7.17	7.23	
Very good	15.25	20.51	
Good	34.98	39.11	
Fair	26.91	26.82	
Poor	15.70	6.34	
Diabetes (%)			< 0.01
Yes	31.84	14.64	
No	65.02	82.0	
Borderline	3.14	3.29	
Unclear	0	0.07	
Congestive heart failure (%)			< 0.01
Yes	14.35	1.95	
No	84.30	97.90	
Unclear	1.35	0.16	
Liver conditions (%)			< 0.01
Yes	11.66	5.19	
No	87.44	94.52	
Unclear	0.90	0.29	
Prescription medicine used (%)			< 0.01
Yes	90.58	57.96	
No	9.42	41.88	
Unclear	0	0.16	

Mean + SD for continuous variables: *P* value was calculated by weighted linear regression model. % for Categorical variables: *P* value as calculated by weighted chi-square test

*BMI* body mass index, *PIR* income to poverty ratio

The analysis of clinical characteristics was shown in Table 2, which revealed that COPD participants had lower blood manganese levels but higher liver stiffness (*P* < 0.05). In the comparison of biochemical indicators between the two groups, COPD participants did not show higher results in liver damage indicators, but indicators related to kidney damage were significantly higher than those of non-COPD participants.

**Table 2:** Characteristics of participants clinical outcomes

Characteristics	COPD (*n* = 223)	Non-COPD (*n* = 4467)	*P* value
Blood Mn (ug/L)	9.42 ± 3.43	9.96 ± 3.72	0.03
Median liver stiffness (kPa)	6.55 ± 4.35	5.98 ± 5.28	< 0.001
HDL (mg/dL)	52.20 ± 16.14	53.16 ± 15.33	0.21
LDL (mg/dL) (%)			< 0.05
< 120	37.67	209.46	
≥ 120	12.11	17.62	
Unclear	50.22	52.92	
ALT (IU/L)	19.83 ± 12.31	22.60 ± 16.59	< 0.05
AST (IU/L)	21.96 ± 15.52	22.07 ± 12.55	0.32
GGT (IU/L)	35.73 ± 44.85	32.29 ± 45.48	0.057
LDH (IU/L)	161.32 ± 30.78	158.84 ± 33.46	0.065
TC (mg/dL)	176.56 ± 37.82	188.98 ± 40.52	< 0.001
TBIL (mg/dL)	0.44 ± 0.26	0.46 ± 0.27	0.156
TP (mg/dL)	7.00 ± 0.47	7.16 ± 0.43	< 0.001
BUN (mg/dL)	17.82 ± 8.95	14.95 ± 5.76	< 0.001
Cr (mg/dL)	0.99 ± 0.38	0.90 ± 0.47	< 0.001

Mean + SD for continuous variables: *P* value was calculated by weighted linear regression model

% for Categorical variables: *P* value as calculated by weighted chi-square test

*HDL* high density lipoprotein, *LDL* low density lipoprotein, *ALT* alanine aminotransferase *AST* Aspartate aminotransferase *GGT* glutamyl transpeptidase, *LDH* lactate dehydrogenase, *TC* blood total cholesterol, *TBIL* blood total bilirubin, *TP* blood total protein, *BUN* blood urea nitrogen, *Cr* blood creatinine

**Fig. 1:**
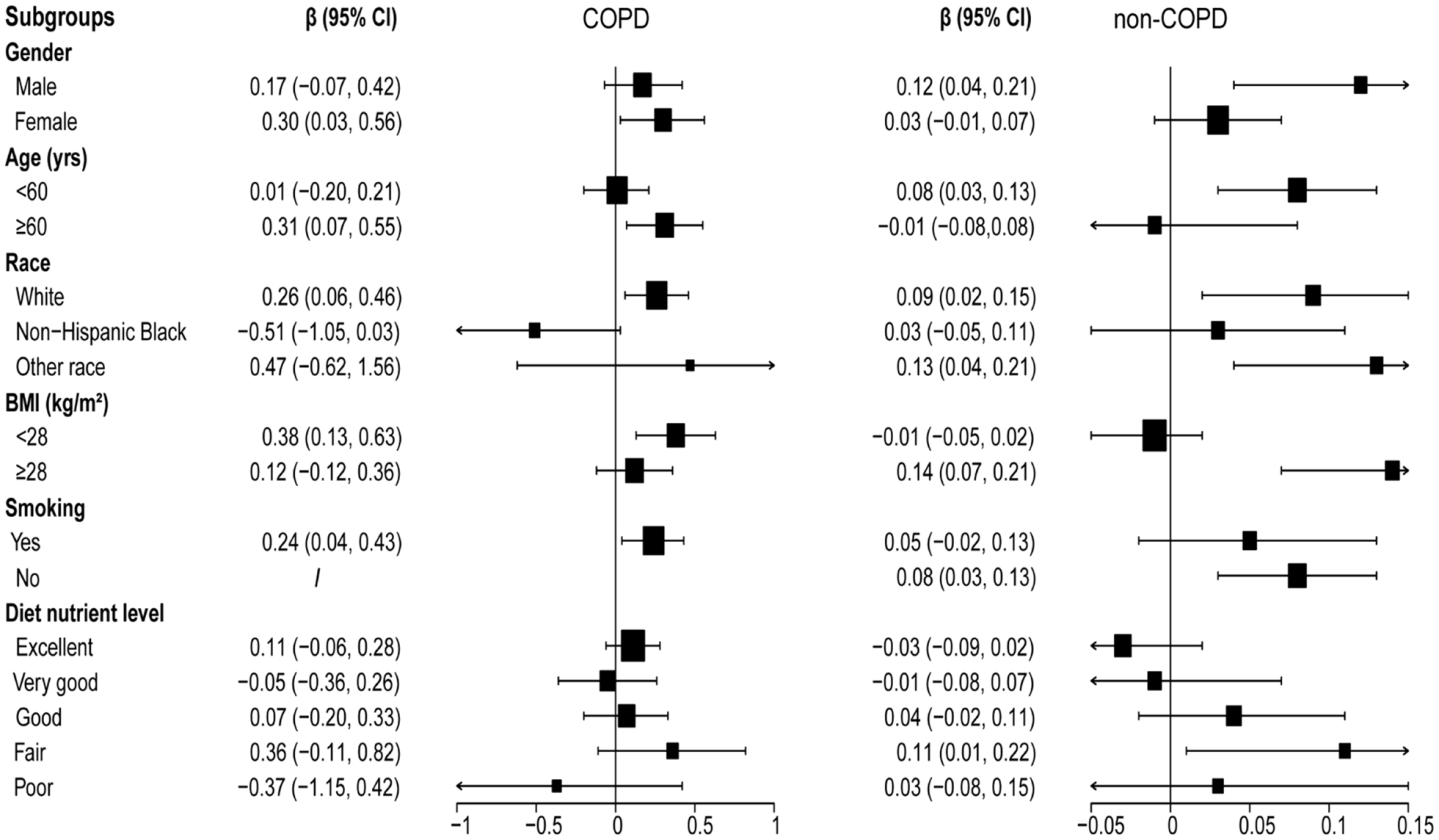
Subgroup analysis of the association between blood manganese and liver stiffness. '/' represented restricted sample size. All the covariates in Table 1 were adjusted. *In the subgroup analysis stratified by each covariate, the model is not adjusted for the stratification variable itself. *BMI* body mass index (kg/m^2^)

Blood manganese was positively associated with liver stiffness in all patients (*β* = 0.08, 95% CI 0.03, 0.12), and this trend was more pronounced in COPD participants (*β* = 0.25, 95% CI 0.08, 0.42) (Table 3). In subsequent subgroup analyses, on the basis of fully adjusted models, the statistically different sensitive crowds in the COPD group were female, age ≥ 60 years, white race, BMI < 28 kg/m [2], and smoking. Non-COPD participants were characterized as male, age < 60 years, other race, BMI ≥ 28 kg/m^2^, non-smoker, and fair diet status (Table 4, Fig. 1).

**Table 3:** Association between blood manganese and liver stiffness

Participants	Model 1	Model 2	Model 3
COPD	0.34 (0.16, 0.51)	0.36 (0.18, 0.54)	0.25 (0.08, 0.42)
Non-COPD	0.10 (0.06, 0.14)	0.14 (0.10, 0.19)	0.07 (0.03, 0.11)
Total	0.11 (0.07, 0.15)	0.15 (0.11, 0.19)	0.08 (0.03, 0.12)

Model 1: No covariates were adjusted. Model 2: age, gender, race were adjusted. Model 3: all the covariates in Table 1 were adjusted

**Table 4:** Subgroup analysis of association between blood manganese and liver stiffness

Characteristics	Model 1		Model 2		Model 3	
	COPD	Non-COPD	COPD	Non-COPD	COPD	Non-COPD
Gender						
Male	0.30 (0.09, 0.52)	0.18 (0.09, 0.26)	0.30 (0.07, 0.53)	0.18 (0.09, 0.27)	0.17 (-0.07, 0.42)	0.12 (0.04, 0.21)
Female	0.36 (0.08, 0.63)	0.07 (0.03, 0.12)	0.41 (0.13, 0.69)	0.10 (0.05, 0.15)	0.30 (0.03, 0.56)	0.03 (-0.01, 0.07)
Age (yrs)						
< 60	0.19 (− 0.03, 0.41)	0.13 (0.08, 0.19)	0.21 (− 0.01,0.44)	0.17 (0.12, 0.23)	0.01 (− 0.20, 0.21)	0.08 (0.03, 0.13)
≥ 60	0.44 (0.20, 0.68)	− 0.01 (− 0.09, 0.07)	0.45 (0.07, 0.55)	0.01 (− 0.08, 0.09)	0.31 (0.07, 0.55)	− 0.01 (− 0.08,0.08)
Race						
White	0.36 (0.16, 0.57)	0.09 (0.03, 0.16)	0.36 (0.16, 0.57)	0.13 (0.07, 0.19)	0.26 (0.06, 0.46)	0.09 (0.02, 0.15)
Non-Hispanic Black	− 0.09 (− 0.62, 0.45)	0.05 (− 0.03, 0.12)	− 0.26 (− 0.82, 0.31)	0.08 (− 0.01, 0.16)	− 0.51 (− 1.05, 0.03)	0.03 (− 0.05, 0.11)
Other race	0.40 (− 0.26, 1.07)	0.12 (0.03, 0.21)	0.42 (− 0.31, 1.14)	0.18 (0.09, 0.27)	0.47 (− 0.62, 1.56)	0.13 (0.04, 0.21)
BMI (kg/m^2^)						
< 28	0.53 (0.28, 0.78)	− 0.02 (− 0.06, 0.01)	0.55 (0.29, 0.81)	0.01 (− 0.03, 0.04)	0.38 (0.13, 0.63)	− 0.01 (− 0.05, 0.02)
≥ 28	0.14 (− 0.10, 0.38)	0.12 (0.05, 0.19)	0.15 (− 0.10, 0.39)	0.16 (0.09, 0.24)	0.12 (− 0.12, 0.36)	0.14 (0.07, 0.21)
Smoking						
Yes	0.39 (0.20, 0.58)	0.11 (0.04, 0.18)	0.42 (0.21, 0.62)	0.15 (0.08, 0.23)	0.24 (0.04, 0.43)	0.05 (− 0.02, 0.13)
No	− 0.07 (− 0.40, 0.27)	0.10 (0.04, 0.15)	0.04 (− 0.28, 0.36)	0.14 (0.09, 0.20)	/	0.08 (0.03, 0.13)
Diet nutrient level						
Excellent	0.15 (− 0.10, 0.39)	− 0.05 (− 0.11, 0.01)	0.05 (− 0.39, 0.48)	− 0.03 (− 0.10, 0.03)	0.11 (− 0.06, 0.28)	− 0.03 (− 0.09, 0.02)
Very good	0.09 (− 0.15, 0.34)	− 0.01 (− 0.08, 0.07)	0.18 (− 0.09, 0.45)	0.01 (− 0.06, 0.09)	− 0.05 (− 0.36, 0.26)	− 0.01 (− 0.08, 0.07)
Good	− 0.03 (− 0.29, 0.23)	0.12 (0.04, 0.19)	− 0.04 (− 0.31, 0.23)	0.14 (0.07, 0.22)	0.07 (− 0.20, 0.33)	0.04 (− 0.02, 0.11)
Fair	0.79 (0.41, 1.17)	0.15 (0.04, 0.26)	0.91 (0.49, 1.32)	0.24 (0.13, 0.35)	0.36 (− 0.11, 0.82)	0.11 (0.01, 0.22)
Poor	− 0.32 (− 0.82, 0.18)	0.03 (− 0.11, 0.16)	− 0.37 (0.93, 0.19)	0.11 (− 0.03, 0.25)	− 0.37 (− 1.15, 0.42)	0.03 (− 0.08, 0.15)

Model 1: no covariates were adjusted. Model 2: age, gender, race were adjusted. Model 3: all the covariates in Table 1 were adjusted

^*^In the subgroup analysis stratified by each covariate, the model is not adjusted for the stratification variable itself

'/' represented restricted sample size

**Fig. 2:**
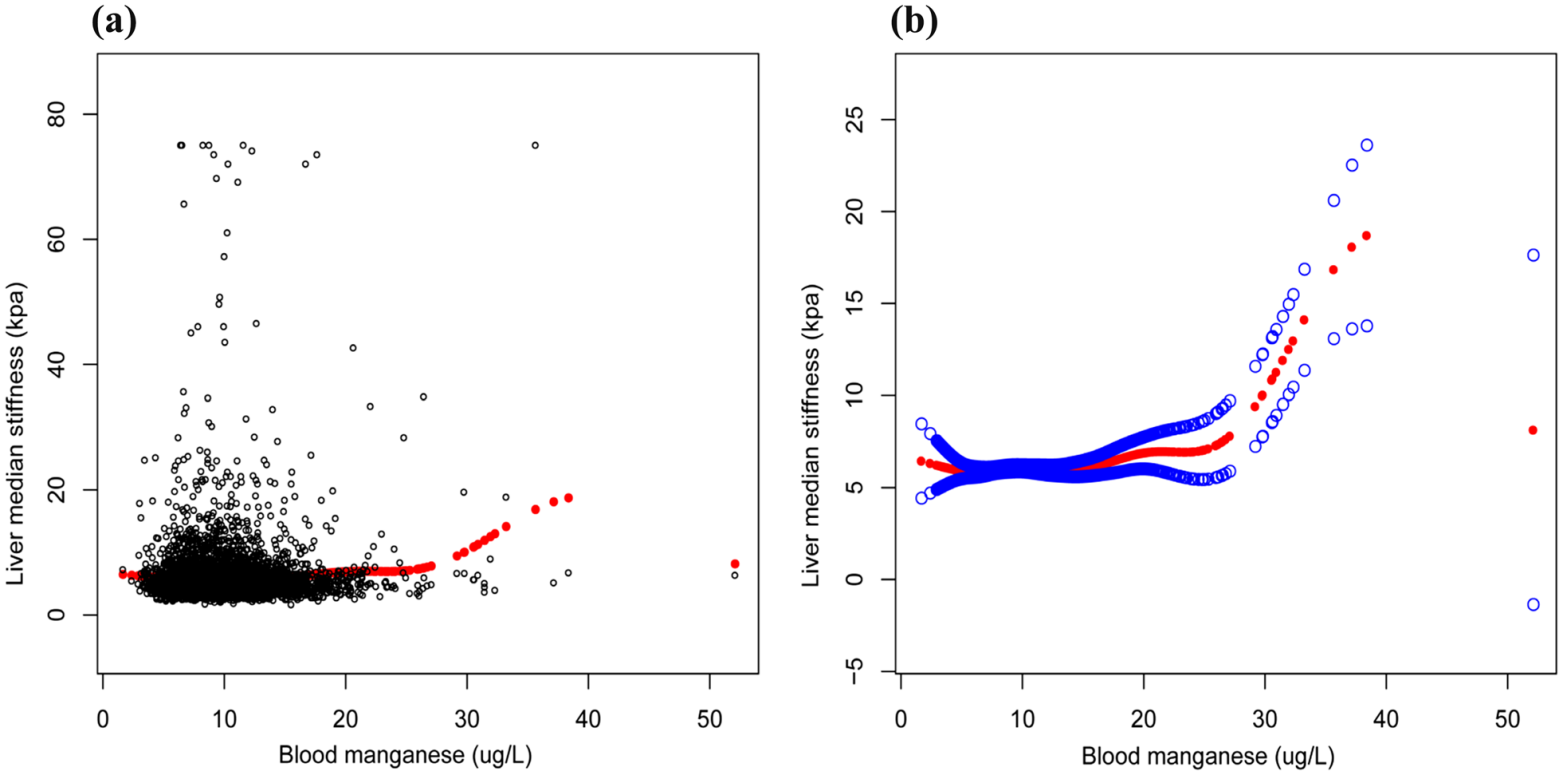
The association between blood manganese and liver stiffness in all participants. **a** Each black point represents a sample. **b** Solid rad line represents the smooth curve fit between variables. Blue bands represent the 95% of confidence interval from the fit. All the covariates in Table 1 were adjusted

**Fig. 3:**
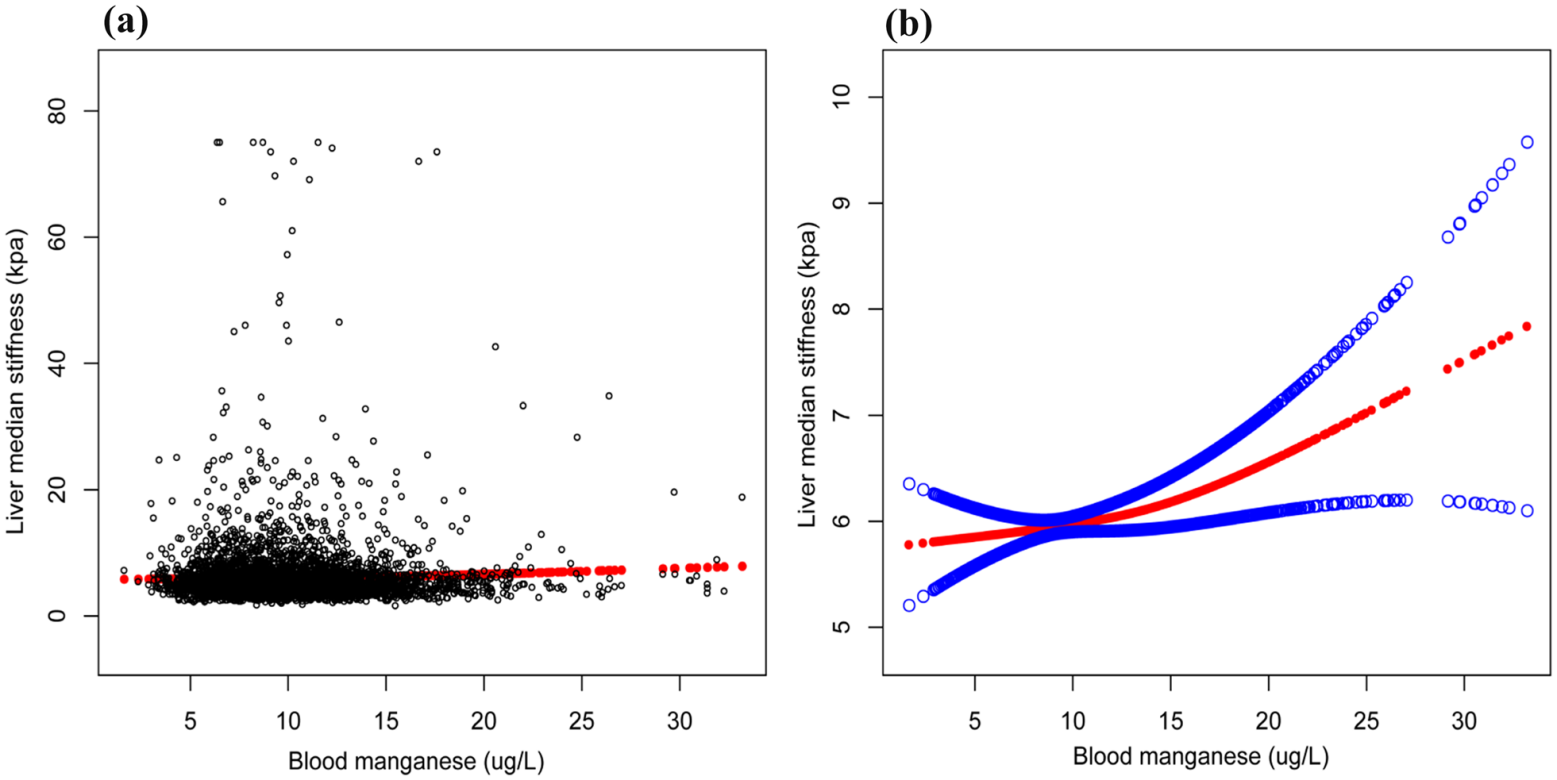
The association between blood manganese and liver stiffness in participants (serum manganese 0-35ug/L). **a** Each black point represents a sample. **b** Solid rad line represents the smooth curve fit between variables. Blue bands represent the 95% of confidence interval from the fit. All the covariates in Table 1 were adjusted

**Fig. 4:**
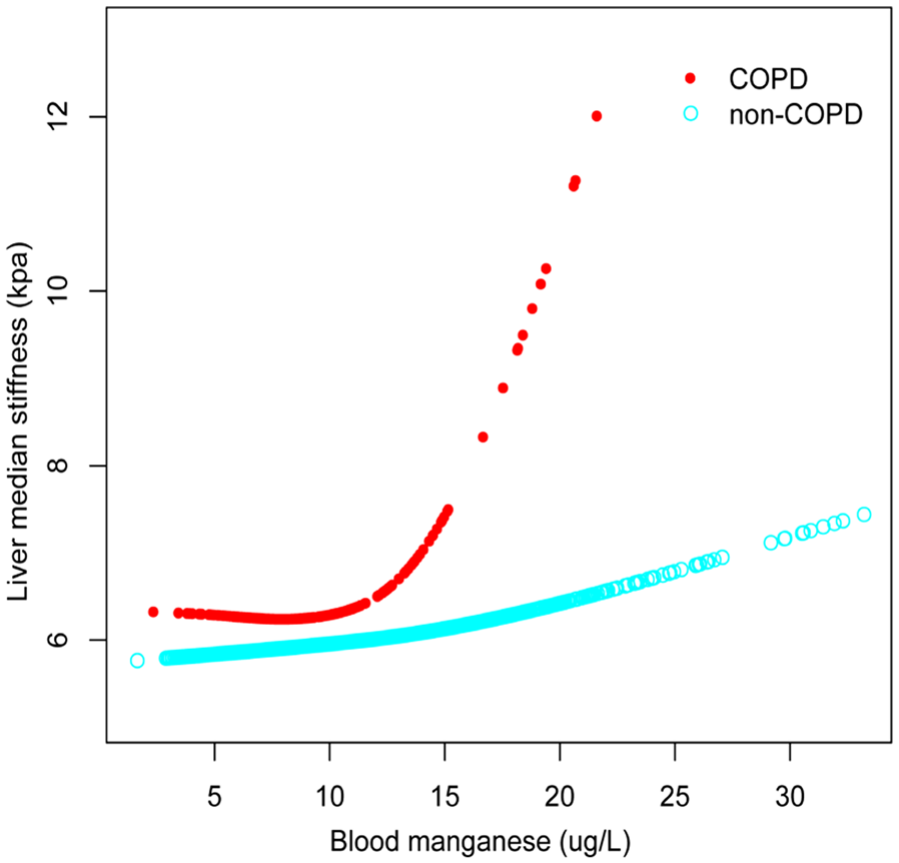
The association between blood manganese and liver stiffness in COPD and non-COPD participants (blood manganese 0-35ug/L). All the covariates in Table 1 were adjusted

To verify whether there was a non-linear relationship between blood manganese and liver stiffness, we performed a smoothed curve fitting analysis (Fig. 2). After excluding significant outliers we set the blood manganese level to 0-35ug/L (Fig. 3) and stratified according to the presence or absence of COPD, showing that the non-linear correlation was more pronounced in the COPD group (Fig. 4).

Subsequently, we performed a threshold effects analysis, and the model selection rule was to choose a non-effect model when the log-likelihood ratio (LLR) was < 0.05, otherwise a linear effects model was selected. Threshold effects analysis suggested a significant increase in the association between blood manganese and liver stiffness when blood manganese exceeded 14.43ug/L in participants with COPD (*β* = 1.76, 95% CI 1.10, 2.41), and a linear effects model was chosen for non-COPD participants (Table 5).

**Table 5:** Threshold effect analysis of association between blood manganese and liver stiffness

Participants	COPD	Non-COPD	Total
Linear effect model	0.24 (0.06, 0.41)	0.07 (0.03, 0.12)	0.08 (0.04, 0.12)
Nonlinear effect model			
Cut-off points (K)	14.43	7.87	13.04
Effect of < K	− 0.04 (-0.24, 0.16)	− 0.09 (− 0.28,0.10)	0.03 (− 0.03, 0.10)
Effect of > K	1.76 (1.10, 2.41)	0.10 (0.05, 0.15)	0.16 (0.07, 0.25)
Log-likelihood ratio	< 0.001	0.08	0.04

All the covariates in Table 1 were adjusted. K: cut-off points

## Discussion

The overall aim of this study was to investigate the association between blood manganese and liver stiffness in COPD participants in NHANES data from 2017 to 2018. Similar to the findings of previous studies, there was a positive correlation between blood manganese and liver stiffness [14], but our highlight was the finding that this positive correlation was more pronounced in COPD participants. In parallel, we found characteristics of sensitive populations with positive association between blood manganese and liver stiffness in COPD participants, such as female, age ≥ 60 years, and smoking. More encouragingly, we found a valid blood manganese threshold in COPD participants. This finding suggests that blood manganese is not only associated with liver stiffness, but also has a more specific meaning for COPD patients, facilitating the monitoring of disease progression and lifestyle modification in sensitive cohorts.

Our study found that COPD participants had higher rates of comorbid diabetes, congestive heart failure, and liver conditions. Epidemiological studies have shown that COPD patients have a 40–50% increased risk of developing type II diabetes mellitus (T2DM) [15], and possible underlying mechanisms for this include systemic inflammation, hypoxia and oxidative stress, all of which have been shown to induce hyperglycemia [16]. An investigation found that left ventricular dysfunction was predominantly prevalent in COPD patients in global initiative for chronic obstructive lung disease (GOLD) class III and IV (70%) [17]. In addition, a study on the prevalence of nonalcoholic fatty liver disease (NAFLD) in COPD patients found for the first time that 41% of COPD patients had moderate to severe fatty liver, 37% had critically ill nonalcoholic fatty liver disease (NASH), and 61% were in the fibrosis stage [18], it suggests that NAFLD is more common in COPD patients. An association between blood manganese and these metabolic complications was also observed: in a cohort study, higher blood manganese levels were associated with an increased risk of T2DM and cardiovascular disease [19].

Our study grouping criteria were based on whether or not the participants had COPD, and we first found that liver stiffness was higher in the COPD cohort. The possible reason for this is that COPD can lead to increased right ventricular pressure due to pulmonary hypertension, which eventually leads to liver congestion, stiffness, and subsequently fibrosis and cirrhosis. However, in this study, there was no significant difference in the transaminase index between the two groups. And we know that transaminases are important biomarkers of liver injury and are elevated in severe liver fibrosis and cirrhosis. This may mean that most of the liver injury caused by COPD is in the compensatory stage. Simultaneously, whether there are other causes of this increased liver stiffness without liver function abnormalities leading to other causes is also worth pondering, for example, whether it is related to manganese exposure. Moreover, we found higher indicators of early kidney injury in COPD participants. This might be because COPD patients have a higher probability of comorbid other systemic diseases, such as diabetic vasculopathy and a wasting state in case of infection, which could have an impact on early renal function. Interestingly, patients with COPD have lower blood manganese concentrations, which do not seem to match their higher liver stiffness. However, this is consistent with previous findings that selenium, manganese and zinc concentrations are lower in patients with COPD than in the normal population [20]. Furthermore, it was demonstrated that increased blood manganese was associated with more severe liver injury [21]. This may explain the absence of significant transaminase abnormalities in the COPD population in this study was their lower blood manganese level. Uz E et al. also found that blood manganese levels were significantly lower in smokers than in non-smokers and demonstrated that this may be due to the interaction of toxic elements and trace elements [22].And to the best of our knowledge, smoking is recognized as a serious risk factor for chronic obstructive pulmonary disease. Notably, Fatemat Hassan et al. studied the determination of manganese in lung tissue samples from COPD patients with different GOLD classifications and found that the onset and exacerbation of COPD was associated with large manganese deposits in lung tissue [23], while one study found elevated superoxide dismutase in the alveolar epithelium of smokers [24]. This may indicate an opposite trend of manganese ion concentration in blood and lung tissue in COPD patients. In conclusion, previous studies suggest that a complex metabolic mechanism also exists between liver stiffness, blood manganese levels, manganese content in lung tissue, and manganese superoxide dismutase in lung tissue in COPD patients. Unfortunately, the current database does not include manganese ion concentrations in liver and lung tissues of COPD patients, so it is not possible to argue this hypothesis, but the present study may, to some extent, provide a direction for subsequent studies.

Excessive manganese exposure could cause a severe liver burden, as the liver is the main organ responsible for storing and metabolizing manganese in the body [5]. Manganese toxicity has the potential to induce oxidative stress, exacerbate mitochondrial dysfunction, dysregulate autophagy, promote apoptosis, and ultimately lead to neurodegeneration [1]. Oxidative stress can disrupt the balance between the rate of removal of reactive oxygen metabolites by enzymes and non-enzymatic antioxidants [25]. It had been proposed that oxidative stress plays a role in the pathogenesis of various physiological conditions and many disease processes, especially COPD [26]. Previous studies have consistently concluded that manganese exposure could lead to liver damage through oxidative stress and mitochondrial damage [27]. In addition, in a cohort study of manganese exposed workers in China, ALT and AST levels were found to increase significantly with increasing manganese exposure. Studies on manganese-induced neurodegeneration are more established, and when experimental animals were given an oral excess of manganese ions, there was a significant increase in oxidative stress markers in brain samples, followed by an increase in acetylcholinesterase activity and glutamate concentration, which exacerbated the degree of intracellular oxidative stress, leading to neurodegeneration.

This study first evaluated an epidemiological study of blood manganese and liver stiffness in the COPD cohort. Subsequently, the relationship between blood manganese and liver stiffness was validated after adjusting for the corresponding covariates and identifying sensitive populations of COPD participants and non-COPD participants. In addition, we found a validated blood manganese fold in COPD participants, which has particular implications for public health care efforts. However, there are some limitations of this study. First, this was a cross-sectional study and it was not possible to determine a causal relationship between blood manganese and liver stiffness. Second, although smoking is a high risk factor for COPD patients, it did not exclude the possibility of a large number of smokers among the non-COPD patients included in this study, and this limitation was due to recall bias. Third, BMI and diet are variable covariates, and whether these variables change over time affects the results of this study needs to be confirmed by more subsequent studies. Finally, because of the limited sample size of participants defined with COPD in this study, the current results remain subject to validation by subsequent studies.

## Conclusion

There was a positive association between blood manganese and liver stiffness, and this association was more pronounced in the COPD population. COPD populations with characteristics of female, age ≥ 60 years, white race, BMI < 28 kg/m^2^ and smoking should be closely monitored for changes in blood manganese levels, especially when blood manganese ions exceed 14.43ug/L might have the potential for higher liver stiffness.

## Manuscript formatting

### Permission to reuse and copyright

Figures, tables, and images will be published under a Creative Commons CC-BY licence and permission must be obtained for use of copyrighted material from other sources (including re-published/adapted/modified/partial figures and images from the internet). It is the responsibility of the authors to acquire the licenses, to follow any citation instructions requested by third-party rights holders, and cover any supplementary charges.

## Data Availability

The NHANES datasets analyzed during the current study are publicly available from the National Center for Health Statistics (NCHS) (https://www.cdc.gov/nchs/nhanes/index.htm), except for geographic data (latitude) that are restricted to use through the NCHS Research Data Center (http://www.cdc.gov/rdc/) per NCHS, Centers for Disease Control and Prevention policy.

## References

[1] Gandhi D, Rudrashetti AP, Rajasekaran S (2022). The impact of environmental and occupational exposures of manganese on pulmonary, hepatic, and renal functions. J Appl Toxicol.

[2] Li L, Yang X (2018). The essential element manganese, oxidative stress, and metabolic diseases: links and interactions. Oxid Med Cell Longev.

[3] Chen P, Culbreth M, Aschner M (2016). Exposure, epidemiology, and mechanism of the environmental toxicant manganese. Environ Sci Pollut Res Int.

[4] Chen P, Bornhorst J, Aschner M (2018). Manganese metabolism in humans. Front Biosci.

[5] Thomsen HS, Svendsen O, Klastrup S (2004). Increased manganese concentration in the liver after oral intake. Acad Radiol.

[6] Kobtan AA, El-Kalla FS, Soliman HH, Zakaria SS, Goda MA (2016). Higher grades and repeated recurrence of hepatic encephalopathy may be related to high serum manganese levels. Biol Trace Elem Res.

[7] Zerón HM, Rodríguez MR, Montes S, Castañeda CR (2011). Blood manganese levels in patients with hepatic encephalopathy. J Trace Elem Med Biol.

[8] Neal SL, Zheng W (2015). Manganese toxicity upon overexposure: a decade in review. Curr Environ Health Rep.

[9] Shakeri MT, Nezami H, Nakhaee S, Aaseth J, Mehrpour O (2021). Assessing heavy metal burden among cigarette smokers and non-smoking individuals in Iran: cluster analysis and principal component analysis. Biol Trace Elem Res.

[10] Wang M (2019). Association between long-term exposure to ambient air pollution and change in quantitatively assessed emphysema and lung function. JAMA.

[11] Zhou Y (2021). Environmental and genetic factors in the pathogenesis of COPD in the road-working population. Dis Markers.

[12] Salvi S (2014). Tobacco smoking and environmental risk factors for chronic obstructive pulmonary disease. Clin Chest Med.

[13] Sigrist R, Liau J, Kaffas AE, Chammas MC, Willmann JK (2017). Ultrasound elastography: review of techniques and clinical applications. Theranostics.

[14] Liu X, Shen H, Chen M, Shao J (2022). Clinical relevance of environmental manganese exposure with liver stiffness and steatosis detected by transient elastography in adults. Environ Sci Pollut Res Int.

[15] Song Y, Klevak A, Manson JE, Buring JE, Liu S (2010). Asthma, chronic obstructive pulmonary disease, and type 2 diabetes in the women’s health study. Diabetes Res Clin Pract.

[16] Katsiki N, Steiropoulos P, Papanas N, Mikhailidis DP (2021). Diabetes mellitus and chronic obstructive pulmonary disease: an overview. Exp Clin Endocrinol Diabetes.

[17] Macchia A (2012). Unrecognised ventricular dysfunction in COPD. Eur Respir J.

[18] Viglino D (2017). Nonalcoholic fatty liver disease in chronic obstructive pulmonary disease. Eur Respir J.

[19] Cabral M (2021). Trace element profile and incidence of type 2 diabetes, cardiovascular disease and colorectal cancer: results from the EPIC-potsdam cohort study. Eur J Nutr.

[20] El-Attar M, Said M, El-Assal G, Sabry NA, Omar E, Ashour L (2009). Serum trace element levels in COPD patient: the relation between trace element supplementation and period of mechanical ventilation in a randomized controlled trial. Respirology.

[21] Zhu X, Yang L, He Y, Sun Y, Shi W, Ou C (2020). Liver function of male rats exposed to manganese at different time points. Biol Trace Elem Res.

[22] Uz E, Sahin S, Hepsen IF, Var A, Sogut S, Akyol O (2003). The relationship between serum trace element changes and visual function in heavy smokers. Acta Ophthalmol Scand.

[23] Hassan F (2014). Accumulation of metals in GOLD4 COPD lungs is associated with decreased CFTR levels. Respir Res.

[24] Harju T (2004). Manganese superoxide dismutase is increased in the airways of smokers’ lungs. Eur Respir J.

[25] Sies H (2015). Oxidative stress: a concept in redox biology and medicine. Redox Biol.

[26] Białas AJ, Sitarek P, Miłkowska-Dymanowska J, Piotrowski WJ, Górski P (2016). The role of mitochondria and oxidative/antioxidative imbalance in pathobiology of chronic obstructive pulmonary disease. Oxid Med Cell Longev.

[27] Jiang J, Wang F, Wang L, Xiao J, Guo D (2020). Manganese chloride exposure causes disorder of energy metabolism and induces oxidative stress and autophagy in chicken liver. Biol Trace Elem Res.

